# Increasing prevalence of HIV infection among first time clients in Italian drug treatment services – is it sexual transmission?

**DOI:** 10.1186/s12879-015-0940-x

**Published:** 2015-04-30

**Authors:** Mario Cruciani, Lucas Wiessing, Giovanni Serpelloni, Bruno Genetti, Alessandra Andreotti, Carpignano Iulia, Monica Zermiani, Barbara Suligoi

**Affiliations:** Center of Community Medicine and HIV Outpatient Clinic, Via Germania, 20, Verona, Italy; European Monitoring Centre for Drugs and Drug Addiction (EMCCDA), Cais do Sodré, 1249-289 Lisboa, Portugal; Dipartimento Politiche Antidroga, Presidenza del Consiglio dei Ministri, Via dei Laterani, 34, 00184 Roma, Italy; Istituto Superiore di Sanità (ISS), Viale Regina Elena, 299, 00161 Roma, Italy

**Keywords:** HIV infection, Hepatitis C virus, Injecting drug users, Epidemiology, Sexually transmitted infections

## Abstract

**Background:**

Over the last two decades, the proportion of people who inject drugs among newly reported HIV cases in Italy has been continuously declining. This trend is reflected in the prevalence of HIV infection among problem drug users followed in drug treatment services. We report nationwide trends in the prevalence of HIV and HCV among tested clients in charge to drug addiction services from 2005 to 2011.

**Methods:**

Data on the prevalence of HIV and HCV among drug users from public drug treatment services across Italy were collected and analyzed for the period from 2005 to 2011. Prevalence of HIV and HCV were compared between clients returning to treatment and those entering treatment for the first time, and by gender. Due to the high percentage of missing data, the “inverse probability weight” method was used. Trends in testing uptake were also analysed.

**Results:**

A significant decrease of HIV and HCV prevalence is observed among all PDUs entering treatment (from 14.7% to 11.1% and from 61.6% to 50%, respectively, in 2005–2011). By contrast, among those entering the services for the first time, after an initial decline the prevalence of HIV infection steadily increased in both sexes, from 2.2% in 2009 to 5.3% in 2011. Self-reported injecting rates in this group decreased over time, and in 2011 the proportion reporting drug injecting was lower among new clients than in people returning to services (14.5 vs. 34.4%). We also observed a progressive and significant reduction in HIV and HCV testing in drug treatment services.

**Conclusions:**

Changes in injection practice and type of drugs used, coupled with a concurrent reduction in HCV prevalence, do not support drug injection as the main explanation for an increased HIV transmission in people entering drug treatment services for the first time. While reductions in testing rates raise concerns over data quality, the possibility of increased sexual transmission needs to be considered.

## Background

In some European countries, particularly in East Europe, HIV infection in people who inject drugs (PWID) is re-emerging as a serious public health problem. Since 2001 and through 2011, data on newly diagnosed HIV infections and HIV prevalence suggested falling infection rates in PWID in the European Union [1-3]. Also in Italy the proportion of PWID among those newly diagnosed with AIDS has been continuously decreasing, from 62.4% before 2001 to 17.6% in 2011/12 [4]. This may, at least partly, follow from the increased availability of prevention, treatment and harm-reduction measures, including opiate substitution treatment and needle and syringe programmes [3,5]. Other factors, such as the decline in injecting drug use that has been reported in some countries (e.g., United Kingdom, Norway), may also have played an important role [6,7]. Recent data, however, suggest that there is a continuing potential for outbreaks of HIV infection among PWID in some European countries, such as Greece and Romania [3,8-12].

Drug injecting has been associated with HIV transmission since the beginning of the epidemic in the early 80’ [13]; however the role of non-injection drug use in the spread of HIV is still unclear [14-16]. Drug use by any route (not just injection) can be associated with unsafe sexual practices, potentially putting people at risk for acquiring or transmitting HIV and other sexually transmitted infections. In particular, stimulants, such as cocaine, crack cocaine and methamphetamine, have been linked to high-risk sexual behaviour [17-19]. Importantly, individuals who engage in high-risk sexual practices (e.g. sex workers, men who have sex with men) may have a higher prevalence of drug use than the general population so that drug use may be associated with high HIV prevalence even without being causally linked to high-risk sexual behaviour [19-21].

In Italy, as in many other countries, HIV prevention programmes for problem drug users (PDUs) have mainly focused on HIV transmission through unsafe injecting practices with less attention to sexual risk behaviours. It has been noted that in many countries, including Italy, Netherlands and USA, the prevalence of HIV infection among non-injecting PDUs has increased (with a simultaneous decrease among PWID) and this increase has been correlated with sexual transmission [22-25].

We analyze data obtained from the national network of drug treatment centres in Italy to investigate the potential role of HIV transmission through sexual contact among non-injecting drug users and to identify HIV trends in specific drug-using subgroups. Where available, results of the HIV, HBV and HCV screening tests were also analyzed.

## Methods

There are 550 drug treatment services located throughout Italy, offering free drug treatment, medical care and psychological assistance on an outpatient basis. Attendance is voluntary and, as defined by law, screening for HIV, HBV and HCV should be offered to all new clients entering the drug treatment services and to returning clients. Data are continuously collected in each service using standardised data forms, regarding: the number of new clients and returning clients, gender, substance(s) of abuse and, where available, results of the HIV, HBV and HCV screening tests. Data are sent to the Ministry of Health and to the Antidrug Policy Department in aggregate format on an annual basis.

For the purpose of this study, we used aggregated data on HIV, HCV and HBV prevalence among people registered in drug treatment services. Screening for HIV, HBV and HCV is performed with enzyme-linked immunosorbent assays on venous blood specimens. In case of HIV and/or HCV seropositivity, subjects are referred to an Infectious diseases or Internal Medicine specialist. Where available, results of HBV serology are interpreted in order to detect subjects candidates to vaccination (anti-HBs and anti-HBc negatives) and those with previous HBV vaccine or infection. Prevalence of HIV and HCV were compared between clients returning to treatment and those entering treatment for the first time, and by gender. Trends in testing uptake were analyzed.

Data were analyzed from 2005 to 2011 using annual reports from the Antidrug Policy Department. Utilization of data collected in the study for scientific purposes was approved by the Bioethics Committee of the Department for antidrug policies.To compare proportions, we used the chi-2 test for trend with double-side p-values. Due to the high percentage of missing data on the raw prevalence of HIV and HCV infection, and in order to reduce the impact of the missing data on the final estimates, the “inverse probability weight” method was used [26].

## Results

**Figure 1:**
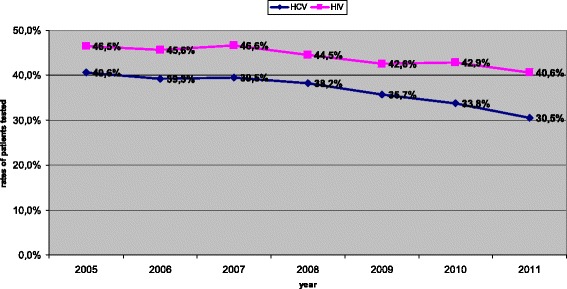
Temporal trends in HCV and HIV screening in Drug addiction services in Italy. A significant decrease in HIV and HCV testing in clients of drug addiction services (p < 0.0001 by χ ^2^ for trends) was observed.

**Figure 2:**
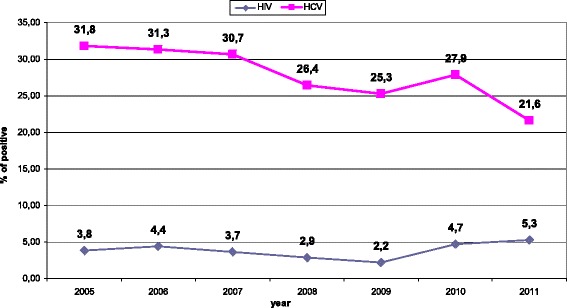
HIV and HCV prevalence among new clients in Drug addiction services in Italy.

From 2005 to 2011 the Italian treatment monitoring system documented between 162,005 and 174,156 clients entering drug treatment services yearly (Table 1). Due to inconsistencies in reporting serologic markers of HBV, data on HBV infection are not reported. We observed a progressive decrease in HIV and HCV screening coverage in drug addiction services, and these differences were statistically significant (p < 0.0001 by χ2 for trend) (Figure 1). The percentage of clients tested for HIV has decreased from 40.6% in 2005 to 30.5% in 2011, and that of clients tested for HCV from 46.5% in 2005 to 40.6% in 2011 (Table 1, Figure 1). Of note, in 2011 only 21.9% of female and 21.1% of male new clients were screened for HIV, with 8.7% (86 women) and 5.5% (340 men) respectively found to be HIV positive.By comparison, in 2005 35.5% of female and 32.4% of male new clients were screened for HIV, with 3.7% of female and 3.3% of male found positive. Due to the high percentage of missing data, the inverse probability weight method was used to analyze data. Overall, prevalence of HIV infection in patients registered in drug addiction services decreased significantly, from 14.7% in 2005 to 11.1% in 2011. However, among those entering the services for the first time (new clients), after an initial decline the prevalence of HIV infection steadily increased in both sexes, from 2.2% in 2009 to 5.3% in 2011 (Table 1, Figure 2).

**Table 1:** **Prevalence of HIV and HCV infection among clients registered in Drug addiction services in Italy**

	**2005**	**2006**	**2007**	**2008**	**2009**	**2010**	**2011** ^**§**^
**HIV prevalence**:							
Overall* *(tested)*	14.7 *(65,848)*	13.5 *(67,300)*	13.1 *(68,032)*	12.9 *(64,021)*	12.0 *(60,057)*	13.8 *(58,408)*	11.1 *(45,663)*
- new clients* *(tested)*	3.8 *(11,562)*	4.4 *(11,520)*	3.7 *(12,165)*	2.9 *(11,214)*	2.2 *(9,821)*	4.7 *(9,860)*	5.3 *(7,154)*
**HCV prevalence**:							
Overall** *(tested)*	61.6 *(75,354)*	62.4 *(78,212)*	61.1 *(80,366)*	59.7 *(74,631)*	59.4 *(71,777)*	61.9 *(74,374)*	50.0 *(56,227)*
- new clients** *(tested)*	31.8 *(11,739)*	31.3 *(11,371)*	30.7 *(12,475)*	26.4 *(11,718)*	25.3 *(10,591)*	27.9 *(10,190)*	21.6 *(8,462)*
Sample size (No. subjects in drug treatment services)	162,005	171,353	172,303	167,674	168,364	174,156	172,211

*p < 0.0001 by χ^2^ for trends, showing a decrease of prevalence of HIV infection in the overall population, and an increase of prevalence of HIV infection among subjects entering Drug addiction services for the first time.

**p < 0.0001 by χ^2^ for trends, showing a decrease of prevalence of HCV infection in all categories of subjects.

^§^For the year 2011 data are incomplete, since some centres provided only anagraphic data; 172,211 clients entered drug treatment in 2011, but data on HIV and HCV screening test were available from 138,475 subjects only. Due to the high percentage of missing data we used the “inverse probability weight” method.

Data on HCV prevalence are shown in Figure 1 and Table 1. HCV prevalence in patients attending drug treatment services is high, but a steady and significant decline has been observed during the last years both in the overall population and in new clients. HCV infections decreased significantly from 61.6% to 50.0% in the overall population tested (p < 0.0001 by χ^2^ for trends), and from 31.8% to 21.6% in new clients (p <0.0001).

Among registered clients in 2011, 79.8% reported opioids as the primary drug of abuse, followed by cocaine in 12.6% and other substances (mostly cannabis) in 7.6%. A different distribution was identified among new clients: 38.7% reported opioids as the primary drug, 30.8% used cocaine, and 25.4% cannabis. Moreover, drug injection was reported by 14.5% of new clients (42.4% of those reporting opioids as primary drug, and 3.9% of those reporting cocaine) compared to 34.4% (58.1% of opioids users, 7.3% of cocaine users) of overall clients. By comparison the 2005 data show a higher proportion of opioids use (45.7%) and a lower cocaine use (26.5%) in new clients, and an overall higher proportion of injective use (22.8% among new clients, and 67.3% among the overall population).

## Discussion

We report a marked increase in the prevalence of HIV coupled with a decrease of HCV prevalence among people entering drug treatment services for the first time in Italy.

Changes in injection practice and type of drugs used, coupled with a concurrent reduction in HCV prevalence, do not support increases in drug injection risks as an explanation for the increase in HIV transmission in this group. A slightly higher HIV prevalence was observed among women compared to men, possibly due to the low number of women in the sample. However, the proportion of HIV-positive women to HIV-positive men (1:2.5) is similar to that reported in the surveillance system for new HIV diagnoses (1:3) in 2011 [27]. Although our monitoring system has national coverage and as such provides key data on infectious diseases in PDU we identify important weaknesses that need to be overcome to improve their relevance for public health.

Among injecting drug users, HIV is transmitted sexually and parenterally, but HCV is transmitted primarily parenterally [28]. Recent evidence suggests that HIV transmission in European countries (Greece, Romania) reporting new outbreaks of HIV infection among injecting drug users was preceded by increases in the prevalence of the HCV [2]. Rising HCV prevalence may act as an early indicator of increases in injecting risks among injecting drug user populations, possibly before HIV has started to spread [28-30]. Given that the increase in HIV prevalence among the new clients in our data is not associated with an increase in HCV prevalence, our data thus seem not to support increases in injection risks as an explanatory factor for the observed increase in HIV.

Data from the Italian National Institute of Health collected in 2005 in a randomly selected sample of 1330 individuals with problematic drug use (defined according to EMCDDA [31] as ‘injecting drug use or long-duration/regular use of opioids, cocaine and/or amphetamines), attending public drug treatment services, showed that the prevalence of HIV was 14.4% among drug injectors and 1.6% among non-injectors; HCV prevalence was 83.2% among injecting drug users and 22.0% among non-injectors [22]. However, the percentage of individuals tested for HIV in those centres has also decreased significantly, from 40.6% in 2005 to 30.5% in 2011 (p < 0.0001 by χ ^2^ for trends).

The progressive reduction of HIV and HCV testing is also one of our main findings, based on a monitoring system with nationwide coverage of drug addiction population in charge to drug addiction services. Results of a survey conducted in Italy in drug treatment services underline the importance of facilitating access to testing and removing obstacles that can lead to the drug user's refusal to undergo testing [32]. The failure to undergo testing among injectors was associated with a shorter history of drug use (<5 years) and with drug centres in central or southern Italy; these associations were also found among non-injectors, with the addition of low level of education. Other reasons for this decline in testing over time include a progressive reduction of resources for public drug treatment services, and a decreased proportion of drug users undergoing pharmacological-psychological treatment (which involves a more complete monitoring program) [33]. Moreover, main reasons for not undergoing HIV testing were an individual’s refusal and lacking of a blood drawing facility within the service. These results underline the importance of facilitating access to testing, of providing the drug dependency centres with the necessary resources for taking blood samples at the centres themselves, of making access to testing uniform throughout the country, and of removing obstacles that can lead to the drug user's refusal to undergo testing.

In Italy, the government response to preventing infections among PDUs is mainly based on opioid substitution treatment (OST), detoxification and psychosocial interventions, while needle and syringe programs (NSP) are mainly implemented through NGOs in the setting of outreach programmes with the training for PWIDs and coverage is very low. Opioid agonist treatments are associated with reductions in the frequency of opioid use, fewer injections and injection-related risk behaviours and lower rates of HIV/HCV prevalence and incidence [34-36]. On the other hand, progressively less attention has been given to periodic testing for HIV, HBV and HCV [37].

The low percentage of PWID tested for HIV observed in drug treatment services leads to a high proportion of subjects with late HIV diagnosis and presentation (late presenters), correlated with a high probability of transmitting HIV and worse clinical outcome [4,38,39]. In Italy in 2012, PWID showed the highest proportion (61.0%) of late presenters for HIV infection as compared to heterosexuals or men who have sex with men [4]. This finding confirms that HIV testing is not routinely performed on a year basis among PWID but rather at a late stage when HIV-indicator diseases are diagnosed, leading to a higher risk of death among PWID with AIDS [40].

Likewise, screening for HCV in drug services remains of utmost importance, since HCV prevalence among injecting drug users remains high, and the availability of improved HCV treatment is expected to reduce the disease burden. HIV/HCV co-infected drug users remain at increased risk of dying from liver-related death in the HAART era compared with HCV-mono-infected drug users [41]. Moreover, recent reports show that among Italian PWID liver disease is the most frequent non-AIDS related cause of death [41].

Another point that deserves attention is the changing epidemiology of problem drug use. In Europe, treatment demand for stimulants (cocaine and amphetamine-type stimulants) and cannabis have increased over time, in parallel with a decline in treatment demands for opiates [42]. Moreover, it has been suggested that the increasing use of non-injecting drugs, such as recreational drugs, may lead to an increased spread of infections through sexual contact [23,36]. Such developments may partly explain our findings for Italy.

Important limitations exist regarding the quality and completeness of our data. First, the progressive reduction in HIV and HCV testing in drug treatment services is of concern, as it can result in unpredictable biased prevalence rates for viral infections, making prevalence trends more difficult to interpret. Second, until present only aggregate data on HIV prevalence are available, which provide summary information but do not allow more detailed epidemiological analysis, such as stratifying seroprevalence rates by type of client, type of substance used, or by route of drug administration.

## Conclusions

Changes in injection practice and type of drugs used, coupled with a concurrent reduction in HCV prevalence, do not support drug injection as the main explanation for an increased HIV transmission in people entering drug treatment services for the first time. While reductions in testing rates raise concerns over data quality, the possibility of increased sexual transmission needs to be considered. Evaluation of this hypothesis and planning of possible effective interventions for reducing HIV transmission via sexual behaviours among PDUs (irrespective of injective or non-injective use) is required.

## Abbreviations

PWID: People who inject drugs
PDUs: Problem drug users
HIV: Human Immunodeficiency Virus
HCV: Hepatitis C Virus
HBV: Hepatitis B Virus
EMCCDA: European Monitoring Centre for Drugs and Drug Addiction
